# Antisense noncoding mitochondrial RNA-2 gives rise to miR-4485-3p by Dicer processing in vitro

**DOI:** 10.1186/s40659-021-00356-0

**Published:** 2021-10-19

**Authors:** Nicole Farfán, Nicole Sanhueza, Macarena Briones, Luis O. Burzio, Verónica A. Burzio

**Affiliations:** 1 Fundación Ciencia & Vida/Andes Biotechnologies SpA, Santiago, Chile; 2 Faculty of Life Sciences, Universidad Andrés Bello, Santiago, Chile; 3 Present Address: Center for Regenerative Medicine, Faculty of Medicine, Universidad del Desarrollo/Clínica Alemana de Santiago, Santiago, Chile; 4 Present Address: Center for Integrative Biology, Faculty of Science, Universidad Mayor de Chile, Santiago, Chile

**Keywords:** ncRNA, miRNA, Cancer, lncRNA, Dicer, Cloning

## Abstract

**Background:**

The antisense noncoding mitochondrial RNAs (ASncmtRNAs) derive from the mitochondrial 16S gene. Knockdown of these transcripts with chemically-modified antisense oligonucleotides induces proliferative arrest, apoptosis and invasiveness reduction in tumor but not normal cells. One of these transcripts, ASncmtRNA-2, contains the complete and identical sequence of hsa-miR-4485-3p and, upon knockdown of this transcript, there is a strong increase in levels of this miRNA, suggesting ASncmtRNA-2 as a source for miR-4485-3p, which is supported by several evidences from our group and others, in the ex vivo setting.

**Results:**

Here we show that incubation of in vitro-transcribed ASncmtRNA-2 with recombinant Dicer produces RNA fragments corresponding to hsa-miR-4485-3p, showing that Dicer binds to and processes ASncmtRNA-2, strongly supporting the hypothesis that ASncmtRNA-2 acts as a precursor for miR-4485-3p.

**Conclusion:**

The in vitro results presented here strengthen the hypothesis that miR-4485-3p is derived from ASncmtRNA-2 by Dicer processing. Since miR-4485-3p is classified as a tumor suppressor miRNA, this evidence strengthens the application of ASncmtRNA knockdown for cancer therapy.

**Supplementary Information:**

The online version contains supplementary material available at 10.1186/s40659-021-00356-0.

## Background

**Fig. 1:**
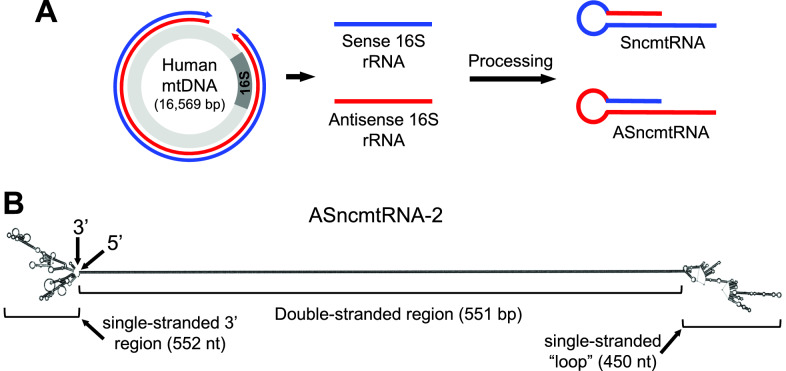
Synthesis and structure of ncmtRNAs. **A** Bidirectional transcription of the mitochondrial genome (red arrow: L-strand transcript; blue arrow: H-strand transcript), giving rise to the Sense (blue) and Antisense (red) transcripts form the 16S rRNA gene. After processing, the chimeric transcripts SncmtRNA and ASncmtRNAs are formed, each with inverted repeats and therefore long dsRNA structural motifs (figure adapted from ref. 4). **B** Predicted secondary structure of ASncmtRNA-2 [5] showing the 552-nt ssRNA 3’ region, the 551-bp dsRNA feature and the 450-nt single-stranded “loop” region

The mitochondrial noncoding RNAs (ncmtRNAs), comprised of Sense (SncmtRNA) and Antisense (ASncmtRNA-1 and ASncmtRNA-2) members, arise from bidirectional transcription of the 16S rRNA gene of the mitochondrial genome (mtDNA) [1, 2]. SncmtRNA contains the complete sense 16S sequence, joined at its 5′ end to a segment from the antisense transcript [1] and the antisense versions are composed of the antisense 16S transcript, with different segments from the sense transcript at its 5′ end [2] (Fig. 1A). Consequentially, these RNAs contain inverted repeats (IRs) and therefore share a distinct structural feature consisting of long double-stranded regions, flanked by a single-stranded “loop” and a single-stranded 3′ region (see Fig. 1B for ASncmtRNA-2 secondary structure, predicted using the ViennaRNA package 2.0 [5]). These transcripts are synthesized inside mitochondria and are exported to the cytosol and nucleus [1, 3].

Knockdown of ASncmtRNAs with chemically-modified antisense oligonucleotides (ASOs) triggers massive apoptotic death in several tumor cell types, preceded by cell cycle arrest, and surviving cells exhibit greatly reduced invasive capacity ex vivo and in vivo [4, 6–9].

An emerging topic in cancer biology is the alteration in levels of many microRNAs (miRNAs) in tumor tissue. In this scenario, some miRNAs are deemed as oncogenic and others as tumor suppressors [10–12]. Several miRNAs derived from the mitochondrial genome have been described [13–15] and one of these, miR-4485-3p, is completely and identically contained in the sequence of the IR of ASncmtRNA-2 [6, 8, 16], suggesting that this lncRNA could constitute a precursor for miR-4485-3p. Indeed, transfection with the ASO Andes-1537, directed to the loop region of the ASncmtRNAs, triggers a nine-fold increase in miR-4485-3p levels [8]. Moreover, transfection of MDA-MB-231 breast cancer cells with miR-4485-3p mimics reduces proliferation [8], suggesting that the effects of the ASO treatment are induced in part by increased expression of miR-4485-3p. We and others have presented strong evidence of the mitochondrial origin of miR-4485 [8, 16] and we showed evidence of an interaction of ASncmtRNA-2 with Dicer by RNA immunoprecipitation followed by RT-PCR [6]. However, whether this interaction translates into Dicer-mediated processing of ASncmtRNA-2 to yield miRNAs had not been determined. Since in vitro interaction and processing studies require in vitro-transcribed RNA from a plasmid containing the complete sequence under study, our research on ncmtRNAs was until recently hindered by cloning difficulties through RT-PCR amplification due to replication slippage of thermostable DNA polymerases on templates with extensive double-stranded structures [17]. Here we present a novel stepwise strategy for cloning this type of transcript with long double-stranded structures. From the resulting clone, we produced in vitro-transcribed ASncmtRNA-2 in order to explore the generation of miR-4485-3p from this transcript. We show that incubation of the transcript with recombinant Dicer in vitro generates a fragment corresponding to this miRNA. This work is an extension of the results we have published previously in an ex vivo setting [8], and strengthens the concept of ASncmtRNA-2 as a precursor for miR-4485-3p.

## Methods

### Cell culture

HEK293T cells (ATCC CRL-3216) were cultured in DMEM (Life Technologies) supplemented with 10% FCS and maintained at 37 °C under a 5% CO_2_-containing atmosphere. Transfections were performed in OptiMEM (Life Technologies).

### ASncmtRNA-2 cloning

**Fig. 2:**
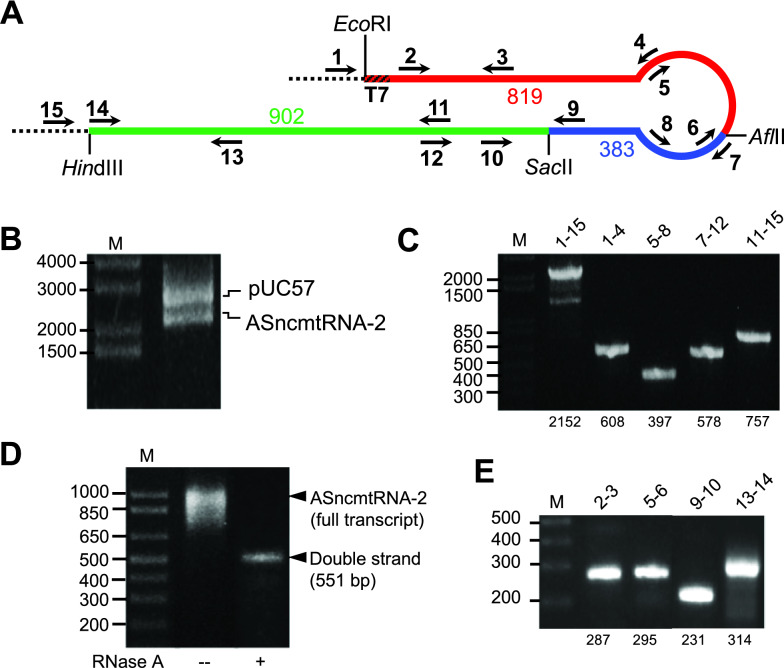
Cloning of ASncmtRNA-2 and in vitro transcription. **A** Schematic diagram of ASncmtRNA-2. The 3 fragments that were joined together for cloning the complete sequence are shown in different colors: fragment 1 (red) was cloned between *Eco*RI and *Afl*II sites and contained a T7 promoter sequence at its 5′ end (crosshatched red); fragment 2 (blue) was cloned between *Afl*II and *Sac*II sites; and fragment 3 (green) was cloned between *Sac*II and *Hin*dIII sites (see also Additional file 1). The size of each fragment is shown in the numbers with the corresponding colors. Arrows and numbers represent the positions of the primers used for PCR (see Additional file 2). The correct and complete cloning of ASncmtRNA-2 was corroborated by *Eco*RI and *Hin*dIII digestion (**B**) and PCR (**C**). **D** The full-length plasmid (pAS2) was linearized and in vitro-transcribed (middle lane). Digestion of the transcript with RNase A yielded a band that migrates slightly above the 500 bp standard, which is consistent with the 551-bp dsRNA region. **E** The correct composition of the transcript was corroborated by RT-PCR. **C**, **E** Amplicon sizes are shown at the bottom of each gel

The complete sequence of ASncmtRNA-2 (GenBank: EU863790.1) was divided into 3 fragments, cloned into the pUC57 vector, obtained by gene synthesis (GenScript; Hong Kong). Figure 2A and Additional file 1 depict the 3 fragments in different colors, starting from the 5’ fragment (red), followed by the middle (blue) and 3’ (green) fragments. Each of these fragments was contained in plasmids pAS2-1, pAS2-2 and pAS2-3, respectively. The insert in pAS2-1 was cloned between the *Eco*RI and *Afl*II restriction sites, and contained a T7 promoter sequence at the 5′ end (Fig. 2A; Additional file 1); pAS2-2 contained the second fragment cloned between *Afl*II and *Sac*II (Fig. 2A; Additional file 1) and pAS2-3 contained the 3′ fragment cloned between *Sac*II and *Hin*dIII (Fig. 2A; Additional file 1). To obtain a plasmid encompassing the complete ASncmtRNA-2 sequence, the cloning procedure was performed in a stepwise manner. First, 2 μg pAS2-3 and 1 μg of pAS2-2 (Additional file 1) were digested with 20 U each of *Sac*II and *Hin*dIII, in a final volume of 100 μl, for 2 h at 37 °C. Reaction mixes were then separated by agarose gel electrophoresis and the pAS2-3 insert and the pAS2-2 plasmid were purified from the corresponding bands. Ligation was performed in a mix containing 200 ng pAS2-3 insert, 200 ng pAS2-2 plasmid (Additional file 1) and 2 U T4 DNA ligase (Promega) in a total volume of 20 μl, at 4 °C for 12 h. Ligation mix was used to transform One shot Max Efficiency DH5α *E. coli* cells (Invitrogen), according to manufacturer’s directions. Plasmid DNA was purified from colonies using the GeneJET Plasmid Miniprep Kit (Thermo Scientific), following manufacturer’s instructions and positive clones were selected by PCR. The resulting plasmid, pAS2-2/3 (1 μg), and pAS2-1 (2 μg) were digested in the manner described above, with *Afl*II and *EcoR*I (Additional file 1). The pAS2-2/3 plasmid and the pAS2-1 insert (Additional file 1) were gel-purified, ligated as above and the ligation mix was used to transform bacteria. The correct insertion and structure of the complete ASncmtRNA2 construction (pAS2; Additional file 1) was corroborated by PCR, restriction analysis and sequencing (Macrogen, South Korea).

### Plasmid PCR

Plasmid pAS2 (50 ng) was amplified by PCR in 25 μl using GoTaq polymerase (Promega). The amplification protocol consisted of an initial 95ºC denaturing step for 10 min, 25 cycles of 95 °C for 30 s, annealing temperature depending on primer Tm (Additional file 2) for 45 s and 72 °C elongation for the time determined by amplicon size (1 min/kbp), and finalizing with a 10 min extension step at 72 °C.

### In vitro transcription

Complete plasmid pAS2 (2 μg) was linearized with HindIII (3′ end of the full transcript) and incubated in a mix containing 10 mM DTT, 80 U RNase-Out, 0,5 mM each rNTP (Promega) and 40 U T7 RNA polimerase (Promega) in the buffer supplied with the enzyme, in a final volume of 100 μl. Polymerization proceeded for 3–6 h at 37 °C. Afterwards, the plasmid was digested with DNA-Turbo (Ambion), according to manufacturer’s directions, and RNA was quantified by UV spectrometry.

### RNase A treatment

Two μg in vitro-transcribed ASncmtRNA-2 was subjected to RNase A digestion using the RNase A kit (Ambion), according to manufacturer’s instructions.

### RT-PCR of in vitro-transcribed RNA

One μg in vitro-transcribed RNA was used for cDNA synthesis in a mix containing 20 pmol specific reverse primer, 3 mM MgCl_2_, 0,5 mM each dNTP, 1 U/μl RNase-Out and 160 U ImProm-II Reverse Transcriptase (Promega) in the buffer supplied with the enzyme, in a final volume of 15 μl. Synthesis was carried out for 5 min at 25 °C, 42 °C for 1 h and 70 °C for 15 min. PCR was performed as described under “Plasmid PCR”, using the primers (IDT) listed in Additional file 2.

### Expression of recombinant Dicer (rDicer)

HEK293T cells (2  ×  10^6^) were seeded into 100-mm petri dishes and transfected the following day with 24 μl LipofectAmine 2000 (Life Technologies) and 12 μg pCMV-DDK-C (Transomic Technologies), which contains the coding sequence for C-terminal FLAG-Tagged wild-type human Dicer1 (pDicer-FLAG). At 24 h, media was switched to DMEM/10% FCS and culture proceeded for another 24 h. Cells were then lysed in 100 μl RIPA (Thermo Scientific), 2 μl PMSF 100X (Sigma-Aldrich) and 2 μl 50X protease inhibitor cocktail (Promega). Mix was incubated on ice with constant pipetting and then centrifuged at 14,000×*g* for 15 min at 4 °C. Dicer-FLAG was immunoprecipitated from the lysate with FLAG Immunoprecipitation kit (Sigma Aldrich), according to manufacturer’s directions. Detection of Dicer-FLAG in the lysate and the immunoprecipitate was confirmed by Western blot as described before [8] (Additional file 3).

### In vitro Dicer processing of ASncmtRNA-2

In vitro-transcribed-ASncmtRNA-2 (3 pmol) was incubated for 15, 30, 45 and 60 min at 37 °C, in a reaction mix containing 30 mM Tris–HCl pH 7.4, 3 mM MgCl_2_, 5 mM DTT, 100 mM KCl, 10% glycerol and 3 pmol rDicer. One mix was prepared without rDicer (T_0_ control). Samples were resolved on a biphasic (top 5% and bottom 15%) denaturing polyacrylamide/8 M Urea electrophoresis alongside microRNA marker and Low Range ssRNA Ladder (New England BioLabs). The gel was transferred to a Nylon membrane (Hybond), which was then UV-crosslinked and pre-hybridized for 30 min at 40ºC in hybridization buffer (HB; 50% Formamide, 5X SSC, 0,1% SDS, 0,2 mg/ml Salmon Sperm DNA and 3X Denhardt’s solution). Membranes were probed with 50 pmol/ml 5′-biotin-labeled probes (IDT) (Additional file 4) in HB for 16 h at 40 °C under constant mixing. Membranes were then washed for 15 min in 2X SSC/0.1% SDS and 15 min in 0.5X SSC, at the same temperature. Biotin label was revealed with the Chemiluminescent Nucleic Acid Detection Module (Thermo Scientific), using a C-DiGit Blot Scanner (Li-Cor Biosciences).

## Results

We previously showed that ASncmtRNA-2 co-immunoprecipitates with Dicer [6], suggesting that this transcript could constitute a substrate for Dicer-mediated processing as a precursor for miR-4485-3p, which is encompassed in the IR of ASncmtRNA-2. In support of this hypothesis, Bianchessi et al. [16] reported that cells treated with ethidium bromide, which precludes mitochondrial transcription [18], induces downregulation of both ASncmtRNA-2 and miR-4485 and, conversely, upregulation of ASncmtRNA-2 brings about an increase in miR-4485 [16].

To test this hypothesis in vitro, we cloned the complete sequence of ASncmtRNA-2 under the control of a T7 promoter. Because of the cloning difficulty presented by the long dsRNA segment (551 bp, see Fig. 1B), we carried out this cloning process in a stepwise manner; 3 successive segments of ASncmtRNA-2 (Fig. 2A; Additional file 1) were cloned into pUC57 (GenScript) and fragments were joined sequentially into a single plasmid (pAS2) encompassing the complete sequence of ASncmtRNA-2. The correct sequence and orientation of the 3 fragments in the complete clone was corroborated by *Eco*RI and *Hin*dIII digestion (Fig. 2B), PCR (Fig. 2C) and sequencing (Macrogen). Full-insert PCR yielded the ASncmtRNA-2 size, but also additional smaller amplicons, probably due to the formation of secondary structures in the template during amplification, which can cause slippage of thermostable DNA polymerases as mentioned [17]. The results show that we successfully cloned the complete sequence of ASncmtRNA-2 using this stepwise strategy, which would be very difficult otherwise. This is especially true for ASncmtRNA-1 which contains a smaller (96-nt) loop region [2], better favoring the fold-back of the IR onto the complementary region in the AS 16S sequence and, in consequence, further hindering a one-step full-sequence cloning strategy. Incidentally, we also managed to successfully clone the full-length ASncmtRNA-1 sequence using the same strategy (N. Farfán, unpublished).

The pAS2 plasmid was used for in vitro transcription with T7 RNA polymerase (Fig. 2D, middle lane). In the native gel, in vitro-transcribed ASncmtRNA-2 migrates close to the 1000 bp dsDNA standard, due to the extensive dsRNA region. The presence of the 551-bp dsRNA motif was corroborated by treatment of the transcript with RNase A (Fig. 2D, right lane). The completeness of the in vitro-generated transcript was verified by RT-PCR (Fig. 2E).

**Fig. 3:**
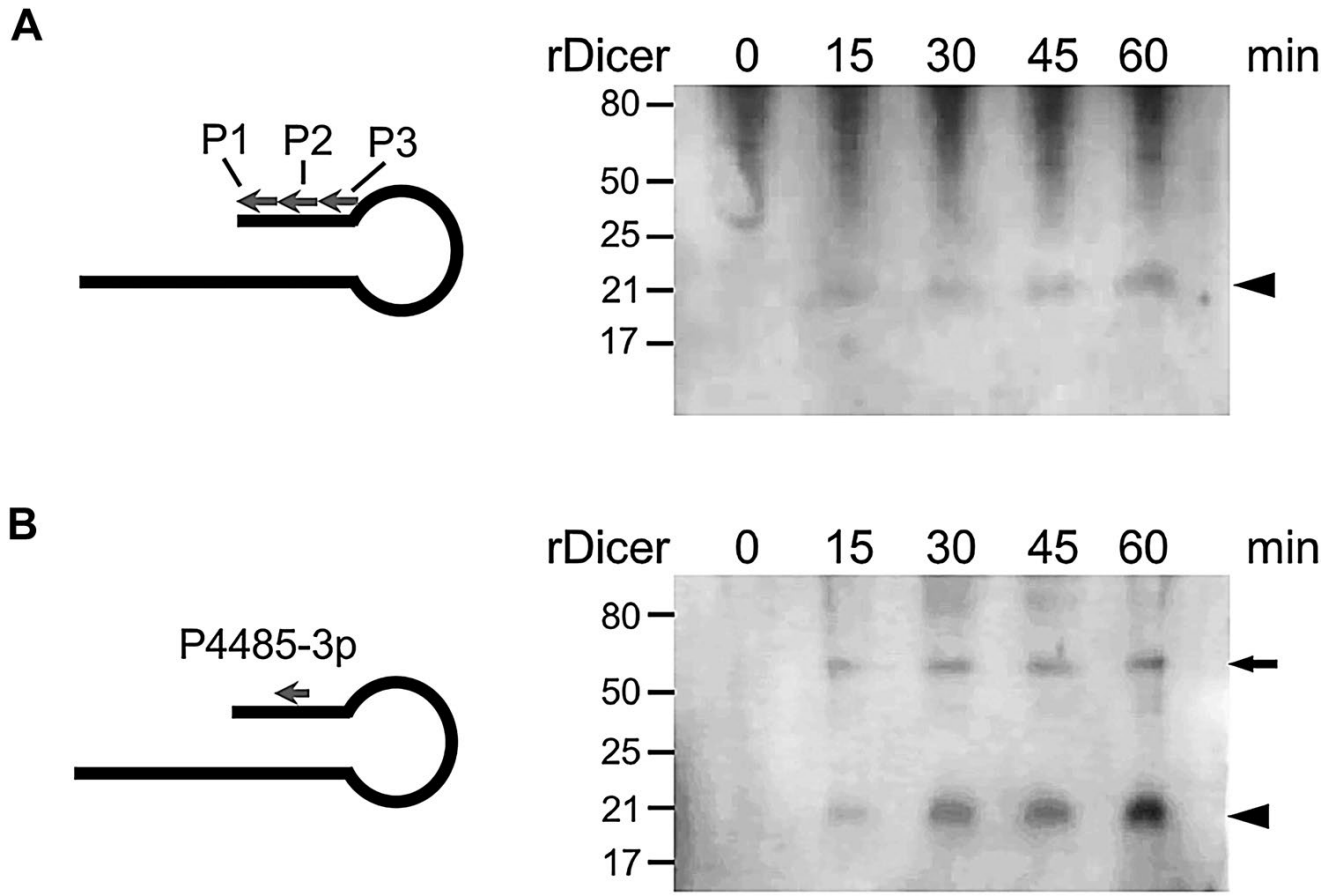
In vitro-transcribed ASncmtRNA-2 is processed by recombinant Dicer in vitro. **A** Transcript was incubated with recombinant rDicer for 0–60 min at 37 °C, after which Northern blot was performed using probes P-AS2a, P-AS2b and P-AS2c (Additional file 4). Arrowhead: putative mature miRNA(s) generated by rDicer processing of the IR. **B** same as **A** but using probe P-4485-3p (Additional file 4). Arrow: pre-miR-4485; arrowhead: mature miR-4485-3p

In order to study the ability of Dicer to process ASncmtRNA-2 in vitro, we expressed wild-type rDicer in HEK293 cells (Additional file 3). We chose not to purchase commercial Dicer enzymes for these experiments since they contain engineered mutations aimed to enhance activity for miRNA functional studies, which could alter the real activity and/or specificity in our experimental setting. The in vitro transcript was incubated with rDicer for 0–60 min and products were analyzed by Northern blot, probed with a mix of 3 biotin-labeled probes (Additional File 4) spanning the complete IR of ASncmtRNA-2 (Fig. 3A). This approach yielded a band that migrated close to the 21-nt RNA standard (Fig. 3A, arrowhead), consistent with the average size of miRNAs. Since this band could hypothetically correspond to or include miR-4485-3p, we then used a probe complementary to this miRNA (see Additional file 4 for sequence and estimated Tm [19]) and we found a defined and more intense band just below the 21-nt standard, consistent with the 20-nt length of miR-4485-3p (miRbase.org). Interestingly, another band was observed between the 50 and 80 standards, which is consistent with the 57-nt length of pre-miR-4485 (miRbase.org). Both bands increased in a time-dependent manner (Fig. 3B). This data shows that rDicer is capable of generating short RNAs of around 20 nt, and that miR-4485-3p and its precursor, pre-miR-4485, are specifically detected.

## Discussion

As mentioned before, knockdown of ASncmtRNAs by Andes-1537 triggers an increase in miR-4485, especially miR-4485-3p [8]. The widely-acknowledged mechanism of ASO treatment relies upon the action of cellular RNase H to cleave the target RNA in the hybrid RNA:DNA duplex segment by hydrolysis of a phosphodiester bond. According to our hypothesis, this cleavage could give rise to an RNA strand, including the IR and part of the ssRNA loop region, that could fold into a pri- or pre-miRNA, which could constitute a substrate for Dicer. So why do we observe processing in vitro without prior RNase H-mediated cleavage? The answer is elusive at the moment and warrants further studies, but we could hypothesize that there is a basal mechanism in the cell that relies on Dicer-mediated processing of the long dsRNA region, in a process similar to the endo-siRNA synthesis mechanism [20]. This first processing step could liberate a ssRNA fragment from the IR containing the pre-miR-4485 which could then be further cleaved by Dicer following the canonical miRNA synthesis pathway, and giving rise to miR-4485-3p. Based on our results, ASncmtRNA-2 could constitute a *bona fide* substrate for Dicer processing in the cell which could account for a basal level of miR-4485-3p, while the knockdown treatment of ASncmtRNA-2 by Andes-1537 could spur a boost in this process. Another possible explanation could involve proteins bound to the ASncmtRNA-2 in the cellular context, which are absent from the in vitro scenario. These are speculations that require further research. Of note, to our knowledge there is no *bona fide* evidence to date on the presence of Dicer in the mitochondrion, so this process would probably occur in the cytosol where the ncmtRNAs are also located [3].

The biological significance of miR-4485 is found as a tumor suppressor in breast and gastric cancer [21, 22]. We showed that this miRNA was upregulated in breast cancer cells after knockdown of ASncmtRNAs [8], which could partly explain the anti-tumoral effect of Andes-1537. We also presented sequence evidence strongly suggesting the mitochondrial origin of pre-miR-4485 [8]. This is not to discard a nuclear origin for this miRNA but it could possibly have a dual origin. Of note, the full mature sequence of miR-4485-5p is also present and in proximity (upstream) of the 3p sequence, but this miRNA increases only modestly after ASncmtRNA knockdown, which led us to hypothesize that it could constitute the passenger strand of the miR-4485 duplex, at least in the mitochondrial-encoded context [8]. The IR also contains the sequence corresponding to miR-1973, which is also upregulated upon ASncmtRNA knockdown. Studies of the possible roles of these mitochondrial-encoded miRNAs are under way. As mentioned above, the ncmtRNAs are synthesized inside mitochondria and exported to the cytosol [3]. Since no hard evidence has been found to date for the presence of Dicer inside mitochondria, the putative miRNA(s) derived from ASncmtRNA-2 are probably Dicer-processed outside the organelle.

## Conclusions

This study provides the first direct evidence of Dicer processing on ASncmtRNA-2, complementing our previous research on the association between ASncmtRNA knockdown and an increase in levels of the tumor suppressor miR-4485-3p [8]. These results shed light upon the molecular mechanisms underlying the selective and widespread effects of ASncmtRNA knockdown on tumor cells.

## Supplementary Information

**Additional file 1: **Stepwise cloning scheme for ASncmtRNA-2.

**Additional file 2: **Sequence of primers (sequence and Tm of primers used for RT and PCR).

**Additional file 3: **Expression of FLAG-tagged recombinant Dicer.

**Additional file 4: **Sequence of probes used for Northern blot.

## Data Availability

The complete sequence of ASncmtRNA-2 can be found at ncbi.nlm.nih.gov (GenBank: EU863790.1).

## Abbreviations

ASncmtRNA: Antisense ncmtRNA
ASO: Antisense oligonucleotide
DMEM: Dulbecco’s modified Eagle’s medium
dNTP: Deoxyribonucleotide triphosphate
dsRNA: Double-stranded RNA
dsDNA: Double-stranded DNA
DTT: Dithiothreitol
FCS: Fetal calf serum
HB: Hybridization buffer
IR: Inverted repeat
lncRNA: Long non-coding RNA
miRNA: MicroRNA
mtDNA: Mitochondrial DNA
ncmtRNAs: Non-coding mitochondrial RNAs
pDicer-FLAG: Plasmid expressing FLAG-tagged human Dicer
rDicer: Recombinant FLAG-tagged human Dicer
RIPA: Radioimmunoprecipitation assay buffer
RNase A: Ribonuclease A
RNase H: Ribonuclease H
rNTP: Ribonucleoside triphosphate
RT-PCR: Reverse transcription coupled to polymerase chain reaction
SncmtRNA: Sense ncmtRNA
ssRNA: Single-stranded RNA
