# Supplementary material for: Antisense noncoding mitochondrial RNA-2 gives rise to miR-4485-3p by Dicer processing in vitro
Source: Biol Res. 2021 Oct 19;54:33. doi: 10.1186/s40659-021-00356-0 (PMC8527801; doi:10.1186/s40659-021-00356-0)
Supplement: Supplementary file 1 — Additional file 1: Stepwise cloning scheme for ASncmtRNA-2. [file 40659_2021_356_MOESM1_ESM.pdf]

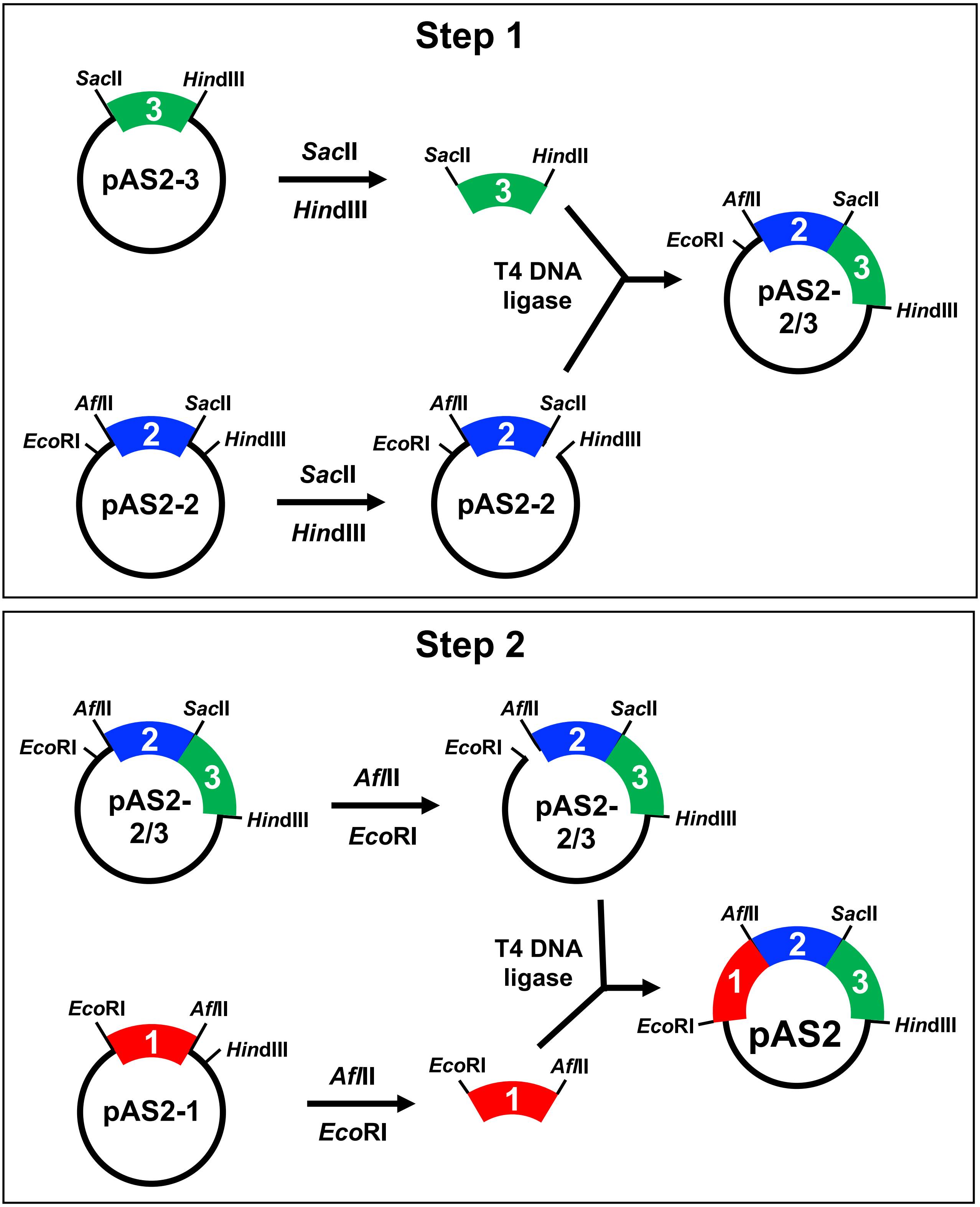

Additional File 1. Stepwise cloning scheme for ASncmtRNA-2. Colors of inserts cloned into pUC57 are the same as the corresponding RNA segments shown in Figure 1A. Only pertinent restriction sites are shown.
