# Supplementary material for: Antisense noncoding mitochondrial RNA-2 gives rise to miR-4485-3p by Dicer processing in vitro
Source: Biol Res. 2021 Oct 19;54:33. doi: 10.1186/s40659-021-00356-0 (PMC8527801; doi:10.1186/s40659-021-00356-0)
Supplement: Supplementary file 2 — Additional file 2: Sequence of primers (sequence and Tm of primers used for RT and PCR). [file 40659_2021_356_MOESM2_ESM.pdf]

Additional File 2: Sequence of Primers

Name	Sequence 5’ – 3’	Tm
1	GTAAAACGACGGCCAG	52
2	TAATACGACTCACTATAGGTACCTAAAAAATCCC	57
3	GGTAAGATTTGCCGAGTTC	54
4	ACCCACCCAAGAACAGG	55
5	CCTGTTCTTGGGTGGGT	56
6	TAGGGATAACAGCGCAATCCTATT	56
7	AATAGGATTGCGCTGTTATCCCTA	57
8	AACCTCCGAGCAGTACATG	56
9	AGTGATTATGCTACCTTTGCACGGT	60
10	GAACTCGGCAAACCTTACC	54
11	GGTTGATTGTAGATATTGGGCT	53
12	AGCCCAATATCTACAATCAACC	53
13	ACCTATAAATCTTCCCACTATTTT	50
14	GGCTAAACCTAGCCCCAAACCC	60
15	CAGGAAACAGCTATGAC	52
