# Supplementary material for: Antisense noncoding mitochondrial RNA-2 gives rise to miR-4485-3p by Dicer processing in vitro
Source: Biol Res. 2021 Oct 19;54:33. doi: 10.1186/s40659-021-00356-0 (PMC8527801; doi:10.1186/s40659-021-00356-0)
Supplement: Supplementary file 3 — Additional file 3: Expression of FLAG-tagged recombinant Dicer. [file 40659_2021_356_MOESM3_ESM.pdf]

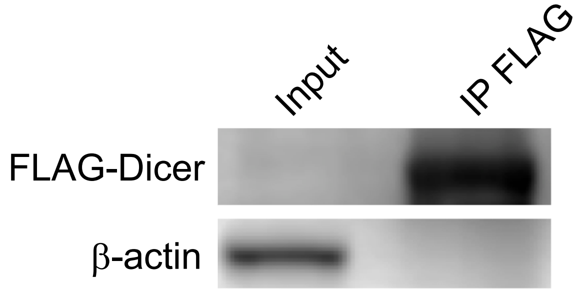

Additional File 3. Expression of FLAG-tagged recombinant Dicer. HEK293T cells were transfected for 48 h with pDicer-FLAG. Lysate was then immunoprecipitated with anti-FLAG antibody and Western blot detection was performed on the immunoprecipitate (IP-FLAG) and the whole lysate (Input), using the anti-FLAG antibody. β-actin was used as internal control.
