# Supplementary material for: Antisense noncoding mitochondrial RNA-2 gives rise to miR-4485-3p by Dicer processing in vitro
Source: Biol Res. 2021 Oct 19;54:33. doi: 10.1186/s40659-021-00356-0 (PMC8527801; doi:10.1186/s40659-021-00356-0)
Supplement: Supplementary file 4 — Additional file 4: Sequence of probes used for Northern blot. [file 40659_2021_356_MOESM4_ESM.pdf]

Additional File 4: Sequence of Probes.

Name	Sequence 5' – 3'
P-AS2a	GGGTTTGTTAGGTACTGTTTGCATTAATAAATTAAAGCTCCATAGGG TCTTCTCGTCTTGCTGTGTCATGCCCGCCTCTTCACGGGCAGGTCA ATTTCACTGGTTAAAAGTAAGAGACAGCTGAACCCTCGTGGAGCCA TTCATACAGGTCCCTATTTAAGGAACAAGTGATTATGCTACCTT
P-AS2b	TTGCACGGTTAGGGTACCGCGGCCGTTAAACATGTGTCACTGGGC AGGCGGTGCCTCTAATACTGGTGATGCTAGAGGTGATGTTTTTGGT AAACAGGCGGGGTAAGGTTTGCCGAGTTCCTTTTACTTTTTTTAACC TTTCCTTATGAGCATGCCTGTGTTGGGTTGACAGTGAGGGTAATAA
P-AS2c	TGACTTGTTGGTTGATTGTAGATATTGGGCTGTTAATTGTCAGTTCA GTGTTTTGATCTGACGCAGGCTTATGCGGAGGAGAATGTTTTCATG TTACTTATACTAACATTAGTTCTTCTATAGGGTGATAGATTGGTCCA ATTGGGTGTGAGGAGTTCAGTTATATGTTTGGGATTTTTTAGGTA
P-4485-3p	TTAGGGTACCGCGGCCGTTA*

*Estimated Tm, calculated as DNA/RNA hybrid in 5X SSC (idtdna.com), and subtracting 2.4 – 2.9ºC per molarity of formamide [19] (11.1 M), is between 39.2 and 44.8ºC.
